# Rolling into the genome: linking mutations to cellular structure through label-free holographic cytometry

**DOI:** 10.1038/s41377-025-02053-z

**Published:** 2025-10-14

**Authors:** Maciej Trusiak

**Affiliations:** Warsaw University of Technology, Institute of Micromechanics and Photonics, Faculty of Mechatronics, Warsaw, Poland

**Keywords:** Interference microscopy, Biophotonics, Imaging and sensing, Phase-contrast microscopy

## Abstract

Holo-tomographic flow cytometry for label-free phenotyping of suspended acute myeloid leukemia blasts is demonstrated. A concave segmentation algorithm is applied to 3D refractive index tomograms to quantify NPM1-mutation-associated cup-like nuclear morphologies, with virtual reality visualization offering engaging immersion. The method enables population-level detection of statistically significant shifts in 3D cell morphology, originally correlating phenotype with genotype.

## Abstract (web summary)

Holo-tomographic flow cytometry allows label-free phenotyping of acute myeloid leukemia blasts, originally correlating phenotype with genotype. A concave segmentation algorithm is applied to 3D refractive index tomograms to quantify NPM1-mutation-associated cup-like nuclear morphologies, with virtual reality visualization offering engaging immersion.

## Graphical abstract

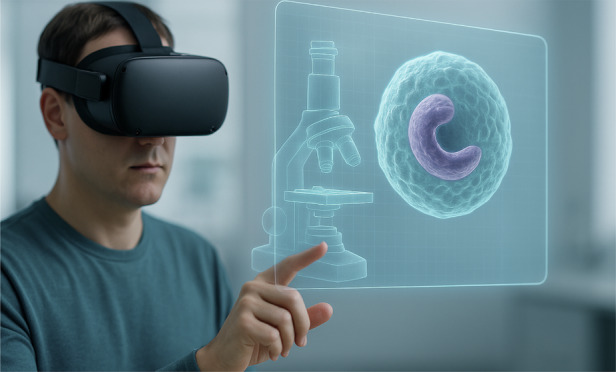

Understanding how genetic mutations influence cellular morphology is essential for advancing diagnostics and treatments, particularly in conditions like acute myeloid leukemia (AML)^1^. In a recent study published in Light: Science & Applications, Pirone and colleagues^2^ extend their signature holo-tomographic flow cytometry (HTFC)^3^ technique to better link genotype to observable cellular phenotypes without labelling.

Fluorescence microscopy remains the gold standard for phenotyping single cells based on molecular labels^4^. However, its reliance on exogenous dyes, extended protocols and limited ability to non-invasively capture intrinsic unimpaired structural properties of live cells^5^ has encouraged the development of complementary label-free approaches^6^. Among them, quantitative phase imaging^7,8^ is of great interest and impact, as it spatially measures optical path variations introduced by refractive index (RI) and physical thickness profiles of the sample. HTFC - based on recording holograms of rotating cells in flow and reconstructing their three-dimensional refractive index map via optical diffraction tomography^9,10^ - offers access to internal morphology without staining^3,11^. In this study^2^, the authors apply HTFC to characterize subtle nuclear shape differences in AML blasts, expanding on their prior work in rolling-cell tomography for nuclear shape detection^12^.

A newly introduced segmentation approach, concave-CSSI (Computational Segmentation based on Statistical Inference)^2^ enables quantitative analysis of nuclei with cup-like morphologies, commonly associated with NPM1 mutations^1^. To improve interpretability of 3D RI data, the authors also integrate virtual reality (VR) environments, enabling immersive exploration of reconstructed tomograms. This adds an intuitive and potentially powerful mode of engaging interaction with, otherwise abstract, high-dimensional biological data and may support educational or clinical applications in the future. Figure 1 shows the sketch of proposed experimental and numerical paths toward AML subcellular-level quantitative label-free immersive examination.

**Fig. 1:**
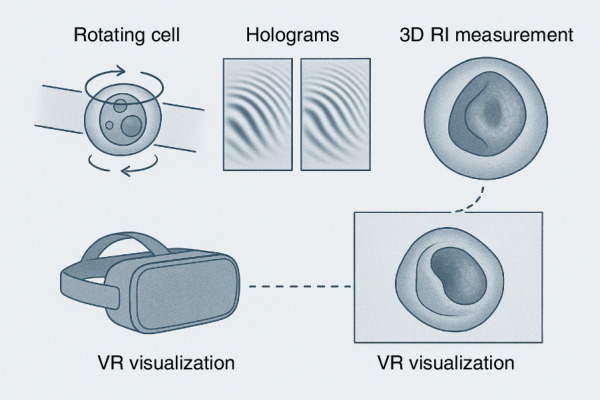
Overview schematic of the proposed label-free HTFC pipeline: leukemic cells flow and rotate within a microchannel under laser illumination; holograms are captured and reconstructed into 3D RI maps, which are further explored via VR to visualize nuclear morphology

The work^2^ is especially notable for demonstrating how 3D RI reconstructions of leukemic blasts phenotype can serve as a morphological fingerprint of a genotype. By analyzing 63 cells per condition across NPM1-mutated and wild-type AML lines, the authors extract a set of quantitative biophysical descriptors that capture statistically significant shifts in cellular architecture. Particularly interesting is the identification and quantification of cup-like nuclear shape - via nucleus sphericity index and normalized nucleus concavity radius - a feature long observed qualitatively in hematopathology but never measured in a label-free, flow-compatible suspended-cell modality. The concave-CSSI algorithm, developed specifically for this application, enables such exotic nuclear segmentation by leveraging voxel-wise RI thresholds and geometric constraints, overcoming limitations of earlier convex-only models^12^.

The study^2^ presents statistically grounded morphological comparisons between populations of AML cells with and without NPM1 mutations, using 3D refractive index reconstructions. These ensemble-level distinctions form a strong foundation for further developments. Future works may focus on enhancing precision toward single-cell classification, particularly for diagnostically ambiguous samples, and broadening the scope of applications. Integrating HTFC with complementary methods - such as correlative fluorescence imaging - could further reinforce its utility and generalizability. Figure 2 highlights the conceptual progression in segmentation capability, from convex to concave nuclei and toward future developments, which might be aimed at organelle-level (e.g., lipid droplets, vacuoles and mitochondria) subcellular segmentation with the potential help of artificial intelligence (AI) and machine learning (ML) based approaches. Similarly, the introduction of standardized “rolling-phantoms” may help consolidate resolution claims and promote adoption across laboratories. These directions, many already acknowledged by the authors, show promising potential for broadening HTFC’s role in quantitative phase cytometry towards widespread clinical applications.

**Fig. 2:**
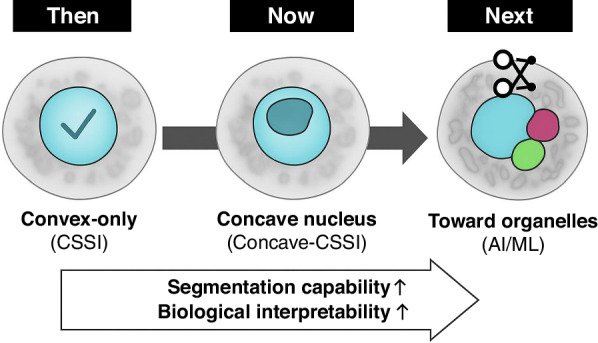
Conceptual diagram showing the evolution of segmentation strategies: from convex nuclei (CSSI)^12^, to concave nuclei (concave-CSSI)^2^, to a future vision of organelle-level (e.g., lipid droplets, vacuoles, mitochondria) segmentation potentially enabled by AI/ML approaches

Overall, the study offers a meaningful step in adapting label-free 3D RI quantitative cytometry to clinically relevant problems. Continued development toward higher nuclear sensitivity, organelle-level precision and direct integration into diagnostic pipelines will further solidify the role of HTFC in next-generation flow cytometry.
